# Design and validation of a questionnaire to measure hantavirus preventive practices in an endemic community

**DOI:** 10.17843/rpmesp.2022.391.9740

**Published:** 2022-03-31

**Authors:** Janeth Agrazal-García, Lydia Gordon-de Isaacs, Ricaurte Tuñón

**Affiliations:** 1 Centro Regional Universitario de Azuero, Universidad de Panamá, Ciudad de Panamá, Panamá. Centro Regional Universitario de Azuero Universidad de Panamá Ciudad de Panamá Panamá; 2 Facultad de Enfermería, Universidad de Panamá, Ciudad de Panamá, Panamá. Facultad de Enfermería Universidad de Panamá Ciudad de Panamá Panamá; 3 Centro Regional Universitario de Coclé, Universidad de Panamá, Ciudad de Panamá, Panamá. Centro Regional Universitario de Coclé Universidad de Panamá Ciudad de Panamá Panamá

**Keywords:** Preventive Medicine, Hantavirus, Surveys and Questionnaires, Validation Study

## Abstract

**Objective.:**

To design and analyze the evidence of content validity, internal structure, and reliability of a questionnaire of preventive practices for hantavirus in an endemic community in the Panamanian context.

**Material and methods.:**

Quantitative study of instrumental design. This research was conducted in four phases: Literature review, content validity through expert judgment with the individual aggregate method and the calculation of the V Aiken, pilot test and psychometric validation, through exploratory factor analysis (EFA) and reliability analysis of instrument scores with ordinal alpha.

**Results.:**

Content validity was evidenced and V Aiken values higher than 0.70 were reported in the lower limit of 95% CI. In the internal structure we identified that the 8 items underlie a single factor that explains 60.70% of the total variance of the test and with factor loadings greater than 0.40; during the reliability analysis, we obtained an ordinal alpha value of 0.84, which is considered good.

**Conclusions.:**

The Hantavirus preventive practical questionnaire is a brief instrument that shows acceptable psychometric properties to measure the activities or behaviors that people carry out to prevent hantavirus.

## INTRODUCTION

Hantavirus is responsible for two syndromes in humans, hemorrhagic fever with renal syndrome (HFRS), mostly found in Eurasia, and hantavirus pulmonary syndrome, more frequent in the Americas ^1^ ^,^ ^2^. The first outbreak of hantavirus in the Americas was identified in 1993 in the United States in the health departments of New Mexico, Arizona, Colorado, and Utah ^3^. Subsequently, cases have been identified in 13 countries in the Americas, including Canada, the United States, Argentina, Bolivia, Brazil, Chile, Ecuador, French Guyana, Paraguay, Peru, Uruguay, Panama, and Costa Rica ^4^.

Hantavirus pulmonary syndrome is an emerging zoonotic disease with a global impact on public health. According to data from the Pan American Health Organization (PAHO), 300 cases are reported each year in the Americas and mortality rates can reach up to 60% ^4^.

Transmission of hantavirus to humans occurs through contact with infected rodents. This virus is excreted in the feces, urine and saliva of asymptomatic infected rodents and is transmitted to humans through inhalation of these aerosols ^2^ ^,^ ^5^. Human exposure to the presence of the infected vector increases with activities in rural areas, forests and agricultural areas, and when the vector is in the household or in the surroundings, becoming risk factors for the development of the hantavirus pulmonary syndrome ^6^.

Preventive practices are the activities or behaviors that people carry out to protect, promote or maintain their health ^7^ and include measures to prevent the onset of the disease and reduce risk factors ^8^. Measuring the practices carried out by people to prevent hantavirus disease is a valuable input that allows us to know how effective past interventions have been; it also serves as evidence to reformulate or restructure future intervention programs and strategies.

During the literature review, we did not identify studies on the design or validation of instruments that measure hantavirus prevention practices and report evidence of psychometric validity. In this sense, this study aims to design and analyze the evidence of content validity, internal structure and reliability of a hantavirus prevention practices questionnaire in an endemic community in the Panamanian context, in order to have a valid and reliable instrument that contributes to provide evidence for the development of prevention programs.

**KEY MESSAGES**

Motivation for the study: To measure the practices or behaviors of people regarding hantavirus prevention is fundamental to propose educational programs and strategies that seek to maintain and/or strengthen adequate practices and improve or restructure inadequate practices. It is important to use an instrument that truly measures what is needed and that complies with a rigorous design.

Main findings: Instrument with eight questions and one dimension that measures hantavirus preventive practices.

Implications for public health: The instrument is valid and reliable to measure hantavirus preventive practices and future studies could integrate observation lists to complement the measurement.

## MATERIALS AND METHODS

### Design of the instrument

An observational study was carried out during ten months, in four specific phases: design of the instrument based on literature review and construct clarity, content validity by expert judgment, pilot test and psychometric validation.

### Literature review and initial development of the instrument

A literature review was conducted in Medline (Pubmed), SciELO and LILACS. The MESH descriptors we used were: hantavirus OR hantavirus pulmonary syndrome; (prevention) OR (preventive measures) AND Americas. The following search strategy was designed for Pubmed: (((hantavirus [Title]) OR (hantavirus pulmonary syndrome [Title])) AND ((prevention) OR (preventive measures [Title])))) AND (Americas), which retrieved 174 documents. We searched for articles with questionnaires measuring hantavirus prevention practices by reviewing the titles and abstracts. We identified three studies ^9^ ^-^ ^11^.

The search in LILACS was based on the following health descriptors (DeCS): hantavirus and prevention, with the following search strategy: TI (hantavirus) AND (prevention), which retrieved seven documents that include guidelines and recommendations on hantavirus and its prevention. Another search was carried out in LILACS with the following strategy: TI (hantavirus) AND (practices) and one study was identified in Argentina ^9^.

The search strategy in SciELO: “hantavirus” AND “practices”, retrieved a study on hantavirus prevention practices along with other zoonoses ^12^. Another study on the effectiveness of public health interventions through behaviors was conducted in New Mexico, Chile and Panama ^13^.

The items were designed and based on the scientific evidence on the mechanisms of hantavirus transmission ^2^ ^,^ ^5^ that support prevention measures and the recommendations of international public health organizations such as PAHO and the Center for Disease Control (CDC) ^14^ ^,^ ^15^; in addition, we reviewed the items elaborated in studies that evaluated hantavirus preventive practices ^9^ ^-^ ^13^.

The identified questionnaires had different validation levels, such as content validity by expert judges, and had been developed by epidemiologists with experience in hantavirus and other tropical diseases, as well as PhD students in Global Health and Microbiology. Another element we considered during the development of the questionnaire was the evidence that the presence of rodent populations is associated with an increased risk of hantavirus pulmonary syndrome ^5^.

Based on this review, we constructed the first preliminary list of 30 questions or items on hantavirus preventive practices and seven sociodemographic and clinical questions, which were submitted to review and discussion by the researchers for a consensus of 20 items on practices and seven sociodemographic and clinical questions (age, sex, monthly family income, educational level, occupation, personal history of hantavirus, history of hantavirus in close relatives, such as parents, children and siblings). The items on practices had polytomous responses (1 to 5) to determine the frequency of the activity (always-never).

### Content validity by expert judgment

Content validity was assessed by means of expert judgment, who are people with vast knowledge of the topic or phenomenon under study, and we used the method of individual aggregates ^16^. A panel of five experts was selected, two of whom were medical epidemiologists with a master’s degree in public health, two nurses with a master’s degree in public health and a doctorate in nursing, and a specialist in health education. Judges had more than 15 years of professional experience; four of the five judges had experience in hantavirus and the other had experience as an expert judge in other public health instruments. The expert judges were provided with the printed and electronic format of the content evaluation template that rates clarity, coherence and relevance, on a scale of one to four ^17^ as well as the the hantavirus preventive practices questionnaire with 20 items. The qualitative assessment of the judges and their quantitative agreement was reviewed through Aiken’s V and its confidence intervals ^18^.

### Pilot test

The purpose of the pilot test was to evaluate the instrument in a population with similar characteristics to the study population, in order to obtain qualitative assessments such as identification of semantic errors, wording and comprehension. Quantitative assessments were included when examining the metric properties of the instrument in its preliminary version ^19^. Based on methodological recommendations ^19^, at the end of the pilot test, the new version of the instrument was presented and reviewed by expert judges for it to be submitted for psychometric validation.

### Psychometric validation

The psychometric validation of the construct was carried out using exploratory factor analysis (EFA), whose main purpose is to explore the structure of the factors that underlie a data set and explain most of the variance of the data set ^20^. The study area for the psychometric validation was the village of El Cacao (second with the highest number of reported cases) in the district of Tonosí, province of Los Santos-Panamá, which has a hantavirus incidence of 681 cases per 100,000 inhabitants ^21^. This town has 315 dwellings; we identified one person over 18 years of age for each dwelling for the application of the questionnaire, preferably the person in charge of the household. We proposed a minimum sample of 200 persons, based on the minimum number of observations necessary for the factor analysis, even in ideal situations of high communalities and well-determined factors ^22^. The participants were selected non-probabilistically by convenience sampling.

The EFA was carried out in three stages. The first stage consisted of preliminary analyses which included reliability analysis and the elimination of items with a corrected item-total correlation lower than 0.30 ^23^; the descriptive analysis of the items (mean, standard deviation, skewness and kurtosis) and the analysis of the feasibility of the EFA, through Bartlett’s statistical test of sphericity, the Kaiser-Mayer-Olkin (KMO) sample adequacy measure and sample adequacy values ^23^. The second stage included the use of the polychoric correlation matrix, which is a suitable option for the EFA when the items are on an ordinal scale ^24^, and it is also recommended when the items are asymmetric with high kurtosis ^25^, as was the case for results. The unweighted least squares method was used for factor estimation, and for the selection of the number of factors to retain, we used the optimal implementation of parallel analysis as well as an oblique rotation (Promin). The unweighted least squares method is highly recommended when working with categorical variables and on the basis of a polychoric correlation matrix ^26^; parallel analysis selects common factors that have eigenvalues greater than those that would be found by chance and is the most appropriate method for evaluating the underlying common factors in variables that are rated on an ordinal scale ^27^. The Promin oblique rotation method provides good results and is simpler to use ^28^. During the third stage, we determined the factor model on the basis of the explained variance, and items with factor loadings equal to or greater than 0.40 were retained ^29^.

For the descriptive analysis and the EFA, we used the freely available FACTOR v11 program designed by Lorenzo-Seva and Ferrando of the University of Tarragona-Spain.

To analyze reliability with the internal consistency of the scores, we used the ordinal alpha which is based on the Excel matrix designed by Domínguez-Lara that uses factor loadings, and is recommended for calculating reliability based on Likert response items ^30^.

Possible interviewer bias was addressed with training for the interviewers and the researcher, which included practical sessions on the application of the questionnaire.

### Ethical Aspects

The study was approved by the research bioethics committee of the Hospital Regional Anita Moreno, Los Santos province, Panama, with the number Proy-38 and with an approval date of February 2020.

## RESULTS

### Content validation

During the qualitative evaluation, three judges made suggestions for improving the phrasing of seven items; two judges recommended omitting the measurements in centimeters and meters from two items: “30 m around the dwelling and 2 cm holes”, since respondents might have difficulty assessing the measurements objectively. After careful review of the judges’ recommendations, the researchers integrated the received suggestions to improve the clarity and phrasing of the items and accepted the recommendation to eliminate the measurements in centimeters and meters.

Table 1 shows the results of the consensus by the expert judges based on Aiken’s V. We found values higher than 0.70 in the lower limit, with a 95% confidence interval, a margin of error of 5% and a mean that ranged from 3.80 to 4.00 on a scale from 1 to 4. Regarding the clarity criterion, six items showed a lower limit of 0.62 (items 3, 4, 15, 16, 18 and 19), and item 12 showed a lower limit of 0.55 in the relevance and coherence criteria. Phrasing was adjusted based on the qualitative recommendations of the judges and in the items with Aiken’s V between 0.62 and 0.55.

**Table 1:** Content validity index (Aiken’s V) and 95% confidence intervals (95% CI) for the criteria of pertinence, coherence and clarity.

Item	Pertinence				Coherence				Clarity			
	Mean	SD	Aiken’s V	95% CI	Mean	SD	Aiken’s V	95% CI	Mean	SD	Aiken’s V	95% CI
1	4.00	0.00	1.00	0.80-1.00	4.00	0.00	1.00	0.80-1.00	3.80	0.45	0.93	0.70-0.99
2	4.00	0.00	1.00	0.80-1.00	3.80	0.45	0.93	0.70-0.99	4.00	0.00	1.00	0.80-1.00
3	4.00	0.00	1.00	0.80-1.00	4.00	0.00	1.00	0.80-1.00	3.60	0.89	0.87	0.62-0.96
4	4.00	0.00	1.00	0.80-1.00	4.00	0.00	1,00	0.80-1.00	3.60	0.55	0.87	0.62-0.96
5	3.80	0.45	0.93	0.70-0.99	3.80	0.45	0.93	0.70-0.99	3.80	0.45	0.93	0.70-0.99
6	3.80	0.45	0.93	0.70-0.99	4.00	0.00	1.00	0.80-1.00	3.80	0.45	0.93	0.70-0.99
7	4.00	0.00	1.00	0.80-1.00	4.00	0.00	1.00	0.80-1.00	4.00	0.00	1.00	0.80-1.00
8	4.00	0.00	1.00	0.80-1.00	4.00	0.00	1.00	0.80-1.00	4.00	0.00	1.00	0.80-1.00
9	4.00	0.00	1.00	0.80-1.00	4.00	0.00	1.00	0.80-1.00	3.80	0.45	0.93	0.70-0.99
10	4.00	0.00	1.00	0.80-1.00	4.00	0.00	1.00	0.80-1.00	3.80	0.45	0.93	0.70-0.99
11	3.80	0.45	0.93	0.70-0.99	3.80	0.45	0.93	0.70-0.99	4.00	0.00	1.00	0.80-1.00
12	4.00	0.00	1.00	0.80-1.00	4.00	0.00	1.00	0.80-1.00	3.40	1.34	0.80	0.55-0.93
13	4.00	0.00	1.00	0.80-1.00	4.00	0.00	1.00	0.80-1.00	4.00	0.00	1.00	0.80-1.00
14	4.00	0.00	1.00	0.80-1.00	4.00	0.00	1.00	0.80-1.00	4.00	0.00	1.00	0.80-1.00
15	4.00	0.00	1.00	0.80-1.00	4.00	0.00	1.00	0.80-1.00	3.60	0.55	0.87	0.62-0.96
16	4.00	0.00	1.00	0.80-1.00	4.00	0.00	1.00	0.80-1.00	3.60	0.55	0.87	0.62-0.96
17	4.00	0.00	1.00	0.80-1.00	4.00	0.00	1.00	0.80-1.00	3.80	0.45	0.93	0.70-0.99
18	4.00	0.00	1.00	0.80-1.00	4.00	0.00	1.00	0.80-1.00	3.60	0.55	0.87	0.62-0.96
19	4.00	0.00	1.00	0.80-1.00	4.00	0.00	1.00	0.80-1.00	3,60	0.55	0.87	0.62-0.96
20	4.00	0.00	1.00	0.80-1.00	4.00	0.00	1.00	0.80-1.00	3.80	0.45	0.93	0.70-0.99

SD: standard deviation; 95% CI: 95% confidence interval.

### Pilot test

The pilot test was applied at the Tonosí Rural Hospital in 32 residents of hantavirus endemic areas in May 2021. The instrument was applied face-to-face by two health professionals who were trained in the application of the instrument, and by the main researcher. Three questions were added to evaluate the comprehension and clarity of the items, as well as the time required to complete the form, and the option of additional suggestions or comments was left. Participants considered the items clear and understandable, the approximate duration was 10-12 min, and some phrasing suggestions were obtained. 

Based on the pilot test we considered the following adjustments: the observations regarding the understanding of each item by the participants and the corrected total item correlation; a value greater than or equal to 0.30 on the total scale was evaluated as the cut-off point; two items that presented low correlations and a certain coincidence with other questions were eliminated; this resulted in a new version with 18 items.

### Psychometric validation

The questionnaire was applied to 213 participants, which represents 67.6% (213/315) of the universe in the study area. Regarding the characteristics of the population (n = 213), 67.8% were women, the age ranged from 18 to 93 years, with a mean of 46 years and a standard deviation of 18.70; 67.8% had an income of less than 300.00 dollars per month; 18.7% had low schooling in (no studies or incomplete primary education). The instrument was applied by the researchers or by a trained collaborator in the participant’s household and the duration ranged between 10-12 min.

First, we carried out a descriptive analysis of the items and response options. Given that only 58.6% (125/213) answered items 13 to 18, which inquired about hantavirus preventive practices related to planting, storing and harvesting food, these were eliminated due to the low response rate, since the community members stated that they were farming for family consumption and did not store the food harvest. We proceeded to work with the remaining 12 items, of which four were eliminated with total corrected item correlation lower than 0.30, prior to an analysis and contrast of the importance of the item in the literature review.

Table 2 shows the descriptive statistics of the adjusted eight-item hantavirus preventive practices instrument; items 2 and 3 had skewness values higher than 3 as absolute value and high kurtosis as well. The percentages of the response options of the items mostly showed high scores, especially item 8, which did not register low scores.

**Table 2:** Descriptive statistics of the items.

Items	Percentage of response options					Metric properties of items			
	1	2	3	4	5	Mean	SD	Skewness	Kurtosis
1	5.6	1.4	12.2	17.8	62.9	4.310	1.106	-1.714	2.243
2	1.9	0.9	3.3	9.4	84.5	4.737	0.737	-3.450	12.760
3	34.7	4.2	11.7	14.1	35.2	3.108	1.727	-0.163	-1.705
4	8.9	0.9	5.2	8.5	76.5	4.427	1.217	-2.102	3.045
5	1.4	0.5	4.7	7.5	85.9	4.761	0.690	-3.482	13.275
6	9.4	0.9	5.6	12.2	71.8	4.362	1.238	-1.945	2.491
7	12.7	1.4	8.0	12.7	65.3	4.164	1.383	-1.495	0.771
8	-	-	8.0	22.5	69.5	4.615	0.631	-1.405	0.805

SD: standard deviation

Table 3 presents the polychoric correlation matrix, which shows low correlations between item 11 and items 3 and 4; the rest of the items have correlations close to 0.30 or higher than 0.40.

**Table 3:** Inter-item polychoric correlations.

	Inter-item correlation							
	Item 1	Item 2	Item 3	Item 4	Item 5	Item 6	Item 7	Item 8
Item 1	1.000							
Item 2	0.258	1.000						
Item 3	0.385	0.273	1.000					
Item 4	0.356	0.401	0.272	1.000				
Item 5	0.356	0.334	0.325	0.522	1.000			
Item 6	0.249	0.424	0.460	0.481	0.501	1.000		
Item 7	0.344	0.479	0.529	0.441	0.484	0.910	1.000	
Item 8	0.448	0.109	0.167	0.445	0.455	0.391	0.321	1.000

We analyzed sample adequacy indices; the KMO value was 0.7565 with a 95% CI of 0.709-0.820, Bartlett’s sphericity test was 833.5 (df = 28; p <0.001) and all items showed simple sample adequacy greater than 0.50, these scores evidence the feasibility of the EFA.

**Figure 1:**
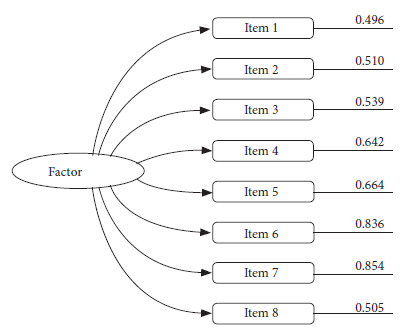
Model and factor loadings of the hantavirus preventive practices instrument.

The unweighted least squares method and the parallel analysis performed in the FACTOR program suggest that the eight items underlie a single factor, which explains 60.70% of the total variance of the test and the factor loadings range from 0.496 to 0.854 as described in Figure 1. The results of the factor loadings are acceptable based on the criteria of retaining items with factor loadings equal to or greater than 0.40.

### Reliability analysis

Reliability was analyzed by means of the ordinal alpha internal consistency test, using the factor loadings of the polychoric correlation matrix, this resulted in a score of 0.84, a value considered good. It should be noted that the ordinal alpha value is a measure of internal consistency that refers to the scores obtained with the instrument in the population and not a metric of the instrument itself.

## DISCUSSION

This study was conducted with the aim of designing and validating a hantavirus preventive practices questionnaire, in an endemic context, which included content validity through expert judges, pilot test, construct validity through exploratory factor analysis and reliability measured with ordinal alpha.

We did not identify many instruments that measure hantavirus preventive practices in our context ^9^ ^-^ ^13^. One of the studies ^9^ we identified explains the use of literature review and hantavirus prevention material developed by health institutions for questionnaire design and another one ^12^ reports the use of focus groups for questionnaire design and validation by an expert. None of the studies presented evidence of psychometric validation. 

The hantavirus preventive practices questionnaire applied in an endemic community showed content validity, Aiken’s V values higher than 0.70 and a margin of error of 5%. The exploratory factor analysis showed that the eight items underlie a single factor that explains 60.70% of the variance. Reliability measured through the ordinal alpha based on the factor loadings of the polychoric correlation matrix was adequate (0.84).

The designed and validated instrument (supplementary material) is a contribution to the measurement of hantavirus preventive practices as it could be applied at the baseline for the development of health education programs for hantavirus prevention; furthermore, it could contribute to assess change in the practices or behavior in pre- and post-intervention focused studies. This instrument was designed around the main prevention strategy currently available, which includes household hygiene practices to prevent rodent entry into the home and the safe cleaning of droppings ^15^ ^,^ ^16^, as well as evidence on the relationship between the presence of rodents and increased risk of hantavirus cases ^5^. The general design of this instrument allows its application in other Latin American countries where hantavirus is endemic; however, it is necessary to consider the following limitation: in this questionnaire it was not possible to measure the items related to the sowing, harvesting and storage of grains in the fields near the house, which represent a risk for human contact with the vector and its droppings.

Another limitation of our study is the low response rate obtained for the questions on hantavirus preventive practices, related to sowing, storage and harvesting of agricultural products, which prevented these items from being used in the EFA, these topics could allow a more complete reading of hantavirus preventive practices. Other limitations of the study include the selection of participants by convenience sampling does not allow generalization of the results; the stability of the instrument over time was not verified, so it would be necessary in future studies to explore test-retest reliability. Finally, given that the questionnaire measures preventive practices by self-report, responses could be biased by learning and self-defense, since the participant tends to answer based on acquired knowledge and expected responses. Consequently, in future research the questionnaire could be complemented with qualitative assessments or other data collection techniques such as observation and the use of checklists.

In conclusion, the hantavirus preventive practices questionnaire is an eight-item unidimensional instrument, that showed evidence of content validity, structure and reliability to measure the practices and/or activities or behaviors carried out by people in an endemic community to prevent hantavirus.
