# Supplementary material for: Design and validation of a questionnaire to measure hantavirus preventive practices in an endemic community
Source: Rev Peru Med Exp Salud Publica. 2022 Mar 31;39(1):47–54. doi: 10.17843/rpmesp.2022.391.9740 (PMC11397759; doi:10.17843/rpmesp.2022.391.9740)
Supplement: Supplementary material. — Available in the electronic version of the RPMESP. [file rpmesp-39-01-9740-s001.pdf]

MATERIAL SUPLEMENTARIO

CUESTIONARIO: PRÁCTICAS PREVENTIVAS DE HANTAVIRUS

Propósito: El siguiente cuestionario pretende identificar las prácticas para la prevención de hantavirus que realizan las personas en una comunidad endémica.

INSTRUCCIONES GENERALES

Se le realizará una serie de preguntas sobre las prácticas que usted realiza, en la repuesta que usted señale, el encuestador colocará una (X). No se registrará su nombre ni datos de identificación personal. La información recolectada será confidencial y será utilizada con el propósito exclusivo de la investigación y futura publicación. Con que frecuencia realiza las siguientes prácticas, las opciones de respuesta son: siempre, casi siempre, a veces, casi nunca, nunca.

**PRÁCTICAS PREVENTIVAS DE HANTAVIRUS**	**Siempre (5)**	**Casi siempre (4)**	**A veces (3)**	**Casi nunca (2)**	**Nunca (1)**
1. Con qué frecuencia sella o tapa los agujeros o rendijas de la casa por donde pueden entrar los ratones.					
2. Durante las últimas semanas con qué frecuencia olvido guardar los alimentos que sobraron de las comidas en recipientes con tapa.					
3. Durante la última semana con qué frecuencia volteo o coloco tapas a los envases donde beben agua las mascotas: perros, gallinas y otros.					
4. Durante las últimas semanas con qué frecuencia se cubrió la nariz con un pañuelo o mascarilla, antes de entrar a lugares que estaban cerrados por cierto tiempo, como cuartos, depósitos o galeras.					
5. Durante las últimas semanas con qué frecuencia utilizo desinfectantes o detergentes para realizar la limpieza de la casa.					
6. Durante las últimas semanas con qué frecuencia levantó polvo cuando limpió lugares cerrados y oscuros					
7. Durante las últimas semanas con qué frecuencia humedeció el piso con agua antes de barrer.					
8. Durante la última semana con qué frecuencia eliminó basura y maleza alrededor de la vivienda.					

Datos sociodemográficos y clínicos

1. Edad: _________
2. Sexo: Fem_____ Masc______
3. Ha enfermado usted alguna vez de hantavirus. SI_____ NO________
4. Ha enfermado algún familiar cercano (madre, padre, hermanos, hijos) alguna vez de hantavirus. SI______ NO_____
5. Nivel educativo:
    - Sin educación______
    - Primaria Incompleta ______
    - Primaria Completa (Hasta 6to grado) ______
    - Secundaria incompleta (Hasta el 5to año de secundaria)___
    - Secundaria Completa________
    - Universitaria ____________
6. Ocupación/Trabajo: __________________________________
7. Ingreso económico familiar mensual: Menos de 300.00 _____________________

    300.00-600.00 ________________________

    601.00-900.00________________________

    Más de 900.00________________________

Rev Peru Med Exp Salud Publica
https://doi.org/10.17843/rpmesp.2022.383.9740
