# Supplementary material for: Design and validation of a questionnaire to measure hantavirus preventive practices in an endemic community
Source: Rev Peru Med Exp Salud Publica. 2022 Mar 31;39(1):47–54. doi: 10.17843/rpmesp.2022.391.9740 (PMC11397759; doi:10.17843/rpmesp.2022.391.9740)
Supplement: Translation

# Translation: Diseño y validación de un cuestionario para medir prácticas preventivas de hantavirus en una comunidad endémica

**Authors:** Janeth Agrazal-García, Lydia Gordon-de Isaacs, Ricaurte Tuñón

**Affiliations:** 1 Centro Regional Universitario de Azuero, Universidad de Panamá, Ciudad de Panama, Panamá.; 2 Facultad de Enfermería, Universidad de Panamá, Ciudad de Panama, Panamá.; 3 Centro Regional Universitario de Coclé, Universidad de Panamá, Ciudad de Panama, Panamá.

**Keywords:** Medicina Preventiva, Hantavirus, Encuestas y Cuestionarios, Estudio de Validación

## Abstract

**Objetivo.:**

Diseñar y analizar las evidencias de validez de contenido, estructura interna y fiabilidad de un cuestionario de prácticas preventivas de hantavirus en una comunidad endémica en el contexto panameño.

**Materiales y métodos.:**

Estudio cuantitativo de diseño instrumental. Esta investigación se desarrolló en cuatro etapas: revisión de literatura, validez de contenido a través de juicio de expertos con el método de agregados individuales y el cálculo de la V de Aiken; prueba piloto y validación psicométrica, a través del análisis factorial exploratorio (AFE) y análisis de fiabilidad de las puntuaciones del instrumento con el alfa ordinal.

**Resultados.:**

Se evidenció validez de contenido y se reportó valores de V de Aiken superiores a 0,70 en el límite inferior del intervalo de confianza al 95%. En la estructura interna se identificó que los 8 ítems subyacen a un solo factor que explica el 60,70% de la varianza total de la prueba y con cargas factoriales superiores a 0,40; en el análisis de fiabilidad se obtuvo un valor de alfa ordinal de 0,84, valor considerado como bueno.

**Conclusiones.:**

El cuestionario de práctica preventivas de hantavirus es un instrumento breve que muestra propiedades psicométricas aceptables para medir las actividades o conductas que realizan las personas para prevenir el hantavirus.

## INTRODUCCIÓN

El hantavirus es responsable de dos síndromes en los seres humanos, la fiebre hemorrágica con síndrome renal (FHRS), presente principalmente en Eurasia, y el síndrome pulmonar por hantavirus más frecuente en las Américas ^1^^,^^2^. El primer brote de hantavirus en las Américas fue identificado en 1993 en los Estados Unidos en los departamentos de salud de Nuevo México, Arizona, Colorado y Utah ^3^. Posteriormente se han identificado casos en 13 países del continente americano como: Canadá, Estados Unidos, Argentina, Bolivia, Brasil, Chile, Ecuador, Guyana Francesa, Paraguay, Perú, Uruguay, Panamá y Costa Rica ^4^.

El síndrome pulmonar por hantavirus es una enfermedad zoonótica emergente de impacto global en la salud pública y que, según datos de la Organización Panamericana de la Salud (OPS), cada año se reportan en las Américas 300 casos y las tasas de mortalidad pueden alcanzar hasta el 60%^ (^ ^4^.

La transmisión del hantavirus al ser humano ocurre por el contacto con roedores infectados. Este virus es excretado por las heces, orina y saliva de los roedores asintomáticos infectados y es transmitido a los seres humanos a través de la inhalación de estos aerosoles ^2^^,^^5^. La exposición humana a la presencia del vector infectado se incrementa al desarrollar actividades en zonas rurales, bosques y áreas agrícolas, y cuando el vector se encuentra en el domicilio o peridomicilio, convirtiéndose en factores de riesgo para el desarrollo del síndrome pulmonar por hantavirus^ (^ ^6^^)^.

Las prácticas preventivas son las actividades o conductas que efectúan las personas para proteger, promover o mantener su salud ^7^^) ^e incluyen medidas para prevenir la aparición de la enfermedad y reducir los factores de riesgo ^8^. Medir las prácticas que realizan las personas para prevenir la enfermedad por hantavirus es un insumo valioso que nos permite conocer qué tan efectivas han sido las intervenciones realizadas en el pasado; además se convierte en evidencia para reformular o reestructurar programas y estrategias de intervención futuras.

En la revisión de la literatura no se identifican estudios de diseño o validación de instrumentos que midan las prácticas de prevención de hantavirus y que reporten evidencias de validez psicométrica. En este sentido, la presente investigación tiene como objetivo diseñar y analizar las evidencias de validez de contenido, estructura interna y confiabilidad de un cuestionario de prácticas preventivas de hantavirus en una comunidad endémica en el contexto panameño, con el fin de contar con un instrumento válido y confiable que contribuya a generar evidencia para el desarrollo de programas de prevención.

**MENSAJES CLAVE**

Motivación para el estudio: medir las prácticas o comportamientos que efectúan las personas para la prevención del hantavirus es fundamental para proponer programas educativos y estrategias que busquen mantener y/o fortalecer las prácticas adecuadas y mejorar o reestructurar las prácticas inadecuadas. Es importante que esta medición se realice con un instrumento que realmente mida lo que deseamos y cumpla con rigor su diseño.

Principales hallazgos: instrumento de ocho preguntas y una dimensión que mide las prácticas preventivas de hantavirus.

Implicancias en la salud pública: el instrumento es válido y confiable para medir prácticas preventivas de hantavirus y en futuros estudios se podría integrar listas de observación para complementar la medición.

## MATERIALES Y MÉTODOS

### Diseño del instrumento

Se realizó un estudio observacional durante diez meses, en cuatro fases especificas: diseño del instrumento en base a la revisión de la literatura y claridad del constructo, validez de contenido por juicio de expertos, prueba piloto y validación psicométrica. 

### Revisión de la literatura y elaboración inicial del instrumento

Se realizó una revisión de literatura en Medline (Pubmed), SciELO y LILACS. Los descriptores MESH utilizados fueron: *hantavirus OR hantavirus pulmonary síndrome; (prevention) OR (preventive measures) AND Américas*. Se elaboró esta estrategia de búsqueda para Pubmed: *(((hantavirus [Title]) OR (hantavirus pulmonary syndrome [Title])) AND ((prevention) OR (preventive measures[Title]))) AND (Americas),* que recuperó 174 documentos. A través de la revisión del título y resumen se buscó artículos con cuestionarios que midieran prácticas para la prevención del hantavirus. Se identificaron tres estudios ^9^ ^-^ ^11^. 

En LILACS la búsqueda se realizó en base a los siguientes descriptores en salud (DeCS): hantavirus y prevención, con la siguiente estrategia de búsqueda: TI (hantavirus) AND (prevención), la cual recuperó siete documentos que incluyen guías y recomendaciones sobre el hantavirus y su prevención. Se realizó otra búsqueda en LILACS con la estrategia: TI (hantavirus) AND (prácticas) y se identificó un estudio en Argentina ^9^. 

En SciELO la búsqueda con la estrategia «hantavirus» AND «prácticas» recuperó un estudio sobre prácticas de prevención del hantavirus junto a otras zoonosis ^12^. Otro estudio sobre la efectividad de las intervenciones en salud pública a través de los comportamientos fue realizado en Nuevo México, Chile y Panamá^ (^ ^13^.

Los ítems se elaboraron y fundamentaron en la evidencia científica sobre los mecanismos de transmisión del hantavirus ^2^ ^,^ ^5^ que sustentan las medidas de prevención y las recomendaciones de organismos internacionales en salud pública como la OPS y el Centro de Control de Enfermedades (CDC, por sus siglas en ingles) ^14^ ^,^ ^15^; además, se revisaron los ítems elaborados en estudios que evaluaron las prácticas preventivas de hantavirus ^9^ ^-^ ^13^. 

Los cuestionarios identificados tenian diferentes niveles de validación, como validez de contenido por jueces expertos, y habían sido desarrollados por epidemiólogos con experiencia en hantavirus y otras enfermedades tropicales, así como estudiantes de doctorado en Salud Global y Microbiología. Otro elemento que se consideró en la elaboración del cuestionario fue la evidencia que la presencia de poblaciones de roedores se asocia con el incremento de riesgo de casos del síndrome pulmonar por hantavirus^ (^ ^5^. 

En base a esta revisión se realizó el primer listado preliminar de 30 preguntas, o reactivos de las prácticas preventivas de hantavirus, y siete preguntas sociodemográficas y clínicas, las cuales fueron sometidas a una revisión y discusión de los investigadores para un consenso de 20 reactivos de las prácticas y siete preguntas sociodemográficas y clínicas (edad, sexo, ingreso económico familiar mensual, nivel educativo, ocupación, antecedente personal de hantavirus, antecedente de hantavirus en familiares cercanos, como padres, hijos y hermanos). Los ítems sobre las prácticas tenían respuesta politómica (1 a 5) para determinar la frecuencia de realización de la práctica (siempre-nunca). 

### Validez de contenido por juicio de expertos

La validez de contenido se realizó a través del juicio de expertos, que son personas con un vasto conocimiento en el tema o fenómeno de estudio y se utilizó el método de agregados individuales^ (^ ^16^. Se seleccionó un panel de cinco expertos, de los cuales dos eran médicos epidemiólogos con maestría en salud pública, dos enfermeras, con maestría en salud pública y doctorado en enfermería y un especialista en educación para la salud. Los jueces contaban con más de 15 años de experiencia profesional; cuatro de los cinco jueces contaban con experiencia en hantavirus y el otro con experiencia como juez experto en otros instrumentos del área de Salud Pública. A los jueces expertos se les entregó en formato impreso y electrónico la plantilla de evaluación del contenido que califica claridad, coherencia y pertinencia, en escala de uno a cuatro^ (^ ^17^ y el cuestionario de prácticas preventivas de hantavirus con 20 reactivos. Se revisó la valoración cualitativa de los jueces y su acuerdo de manera cuantitativa a través de la V de Aiken y sus intervalos de confianza ^18^. 

### Prueba piloto

La prueba piloto tuvo como objetivo evaluar el instrumento de medición en una población con características similares a la del estudio, con el fin de obtener valoraciones cualitativas como identificación de errores semánticos, redacción y comprensión. Se incluyó valoraciones cuantitativas al examinar las propiedades métricas del instrumento en su versión preliminar^ (^ ^19^. En base a recomendaciones metodológicas ^19^ al finalizar la prueba piloto se presentó la nueva versión del instrumento que fue revisada por los jueces expertos para la versión que se presentaría a validación psicométrica.

### Validación psicométrica

La validación psicométrica del constructo se hizo empleando el análisis factorial exploratorio (AFE), que tiene como propósito principal explorar la estructura de los factores que subyacen de un conjunto de datos y que explican la mayor parte de la varianza del conjunto de datos^ (^ ^20^. El área de estudio para la validación psicométrica fue el corregimiento de El Cacao (segundo con mayor reporte de casos) del distrito de Tonosí, provincia de Los Santos-Panamá, el cual tiene una incidencia de hantavirus de 681 casos por 100 000 habitantes^ (^ ^21^. Este poblado cuenta con 315 viviendas; se identificó una persona mayor de 18 años por cada vivienda para la aplicación del cuestionario, de preferencia el encargado del hogar. Como muestra mínima se propuso 200 personas en base al número de observaciones mínimas necesarias para el análisis factorial, aun en situaciones ideales de altas comunalidades y factores bien determinados^ (^ ^22^. Los participantes fueron seleccionados de manera no probabilística a través de un muestreo por conveniencia. 

Para la realización del AFE se siguió tres etapas. En el primero se realizaron los análisis preliminares que incluyen el análisis de fiabilidad y se eliminaron los ítems con una correlación ítem-total corregida inferior a 0,30 ^23^; el análisis descriptivo de los ítems (media, desviación estándar, asimetría y curtosis) y el análisis de viabilidad del AFE, a través de la prueba estadística de esfericidad de Bartlett, la medida de adecuación muestral de Kaiser-Mayer-Olkin (KMO) y valores de adecuación muestral ^23^. En el segundo paso se utilizó la matriz de correlaciones policóricas, opción adecuada para el AFE cuando los ítems o reactivos se encuentran en una escala ordinal ^24^, además se recomiendan cuando los ítems son asimétricos con curtosis elevadas ^25^, como ocurrió en estos resultados. Como método de estimación de factores se utilizó el de mínimos cuadrados no ponderados, y para la selección del número de factores a retener se utilizó la implementación óptima de análisis paralelo y se utilizó una rotación oblicua (Promin). El método de mínimos cuadrados no ponderados es muy recomendado cuando se trabaja con variables categóricas y en base a una matriz de correlación policóricas ^26^; el análisis paralelo selecciona factores comunes que presentan valores propios mayores a los que se encontrarían por azar y es el método más apropiado para evaluar los factores comunes subyacentes en variables que se califican en una escala ordinal ^27^. El método de rotación oblicua Promin alcanza buenas soluciones y es más simple de usar ^28^. En el tercer paso se determinó el modelo factorial en base a la varianza explicada y se retuvo los ítems con cargas factoriales iguales o superiores a 0,40 ^29^.

Para el análisis descriptivo y el AFE, se utilizó el programa FACTOR v11 de acceso gratuito diseñado por Lorenzo-Seva y Ferrando de la Universidad de Tarragona-España.

Para el análisis de fiabilidad a través de la consistencia interna de las puntuaciones se utilizó el alfa ordinal en base a la matriz en Excel diseñada por Domínguez-Lara que utiliza cargas factoriales, lo que se recomienda para calcular la fiabilidad en función de los ítems de respuesta Likert ^30^.

El posible sesgo del entrevistador fue abordado a través del entrenamiento de los entrevistadores y el investigador; que incluyó sesiones prácticas de aplicación del cuestionario.

### Aspectos éticos

El estudio fue aprobado por el comité de bioética de investigación del Hospital Regional Anita Moreno, provincia de Los Santos, Panamá, con el número Proy-38 y con fecha de aprobación en febrero del 2020.

## RESULTADOS

### Validación de contenido

En la evaluación cualitativa, tres jueces dieron propuestas de mejora en la redacción de siete ítems; dos jueces recomendaron omitir las medidas en centímetros y metros de dos ítems: «30 m alrededor de la vivienda y agujeros de 2 cm», ya que los encuestados podrían tener dificultades para valorar las medidas de manera objetiva. Después de la revisión cuidadosa de las recomendaciones de los jueces, los investigadores, integraron las propuestas recibidas para mejorar la claridad y redacción de los ítems y se acoge la recomendación de eliminar las medidas en centímetros y metros.

En la Tabla 1 se presentan los resultados del acuerdo de los jueces expertos, en base a la V de Aiken se observan valores superiores a 0,70 en el límite inferior, con un intervalo de confianza al 95% y un margen de error del 5% y una media que osciló de 3,80 a 4,00 en una escala del 1 al 4. Con relación al criterio de claridad, seis ítems presentaron límite inferior de 0,62 (ítems 3, 4, 15, 16, 18 y 19), y el ítem 12 mostró un límite inferior de 0,55 en los criterios pertinencia y coherencia. Se realizaron ajustes en la redacción en base a las recomendaciones cualitativa de los jueces y en los ítems con V de Aiken entre 0,62 y 0,55.

**Tabla 1:** Índice de validez de contenido (V de Aiken) e intervalos de confianza al 95% (IC 95%) en los criterios de pertinencia, coherencia y claridad.

Ítem	Pertinencia				Coherencia				Claridad			
	Media	DE	V de Aiken	IC 95%	Media	DE	V de Aiken	IC 95%	Media	DE	V de Aiken	IC 95%
1	4,00	0,00	1,00	0,80-1,00	4,00	0,00	1,00	0,80-1,00	3,80	0,45	0,93	0,70-0,99
2	4,00	0,00	1,00	0,80-1,00	3,80	0,45	0,93	0,70-0,99	4,00	0,00	1,00	0,80-1,00
3	4,00	0,00	1,00	0,80-1,00	4,00	0,00	1,00	0,80-1,00	3,60	0,89	0,87	0,62-0,96
4	4,00	0,00	1,00	0,80-1,00	4,00	0,00	1,00	0,80-1,00	3,60	0,55	0,87	0,62-0,96
5	3,80	0,45	0,93	0,70-0,99	3,80	0,45	0,93	0,70-0.99	3,80	0,45	0,93	0,70-0,99
6	3,80	0,45	0,93	0,70-0,99	4,00	0,00	1,00	0,80-1,00	3,80	0,45	0,93	0,70-0,99
7	4,00	0,00	1,00	0,80-1,00	4,00	0,00	1,00	0,80-1,00	4,00	0,00	1,00	0,80-1,00
8	4,00	0,00	1,00	0,80-1,00	4,00	0,00	1,00	0,80-1,00	4,00	0,00	1,00	0,80-1,00
9	4,00	0,00	1,00	0,80-1,00	4,00	0,00	1,00	0,80-1,00	3,80	0,45	0,93	0,70-0,99
10	4,00	0,00	1,00	0,80-1,00	4,00	0,00	1,00	0,80-1,00	3,80	0,45	0,93	0,70-0,99
11	3,80	0,45	0,93	0,70-0,99	3,80	0,45	0,93	0,70-0,99	4,00	0,00	1,00	0,80-1,00
12	4,00	0,00	1,00	0,80-1,00	4,00	0,00	1,00	0,80-1,00	3,40	1,34	0,80	0,55-0,93
13	4,00	0,00	1,00	0,80-1,00	4,00	0,00	1,00	0,80-1,00	4,00	0,00	1,00	0,80-1,00
14	4,00	0,00	1,00	0,80-1,00	4,00	0,00	1,00	0,80-1,00	4,00	0,00	1,00	0,80-1,00
15	4,00	0,00	1,00	0,80-1,00	4,00	0,00	1,00	0,80-1,00	3,60	0,55	0,87	0,62-0,96
16	4,00	0,00	1,00	0,80-1,00	4,00	0,00	1,00	0,80-1,00	3,60	0,55	0,87	0,62-0,96
17	4,00	0,00	1,00	0,80-1,00	4,00	0,00	1,00	0,80-1,00	3,80	0,45	0,93	0,70-0,99
18	4,00	0,00	1,00	0,80-1,00	4,00	0,00	1,00	0,80-1,00	3,60	0,55	0,87	0,62-0,96
19	4,00	0,00	1,00	0,80-1,00	4,00	0,00	1,00	0,80-1,00	3,60	0,55	0,87	0,62-0,96
20	4,00	0,00	1,00	0,80-1,00	4,00	0,00	1,00	0,80-1,00	3,80	0,45	0,93	0,70-0,99

DE: desviación estándar; IC 95%: intervalo de confianza al 95%.

### Prueba piloto

La prueba piloto se aplicó en el Hospital Rural de Tonosí en 32 residentes de áreas endémicas de hantavirus, en mayo de 2021. El instrumento fue aplicado de manera presencial por dos profesionales sanitarios que fueron orientadas sobre la aplicación del instrumento, y por la investigadora principal. Se agregaron tres preguntas para evaluar la comprensión y claridad de los ítems, así como el tiempo necesario para completar el formulario y se dejó la opción de sugerencias o comentarios adicionales. Los participantes consideraron los ítems claros y comprensibles, el tiempo de aplicación aproximado fue de 10-12 min, y se obtuvo algunas sugerencias de redacción. 

Para los ajustes en base a la prueba piloto, se tomaron en cuentas las observaciones sobre comprensión del ítem por los participantes y la correlación ítem total corregida; se evaluó como punto de corte un valor mayor o igual a 0,30 en la escala total; se eliminaron dos ítems que presentaron correlaciones bajas y cierta coincidencia con otras preguntas, dando como resultado una nueva versión con 18 ítems.

### Validación psicométrica

Se aplicó el cuestionario a 213 participantes, lo que representa un 67,6% (213/315) del universo en el área de estudio. En cuanto a las características de la población (n = 213) el 67,8% son mujeres, la edad osciló entre 18 y 93 años, con una media de 46 años y una desviación estándar de 18,70, con un ingreso económico inferior a 300,00 dólares mensuales en el 67,8%, y una escolaridad baja en 18,7% (no tienen estudios o realizó una primaria incompleta). El instrumento fue aplicado por los investigadores o por un colaborador entrenado en la vivienda del participante y el tiempo de aplicación osciló entre 10-12 min.

En primer lugar se realizó el análisis descriptivo de los ítems y las opciones de respuesta. Dado que solo el 58,6% (125/213) contestaron los ítems del 13 al 18, que indagaban sobre las prácticas preventivas de hantavirus relacionadas con la siembra, almacenamiento y cosecha de alimentos, estos fueron eliminados por el bajo porcentaje de respuesta ya que los miembros de la comunidad manifestaron que realizaban agricultura para el consumo familiar y no almacenaban la cosecha de alimentos. Se procedió a trabajar con los 12 ítems restantes, de los cuales se eliminaron cuatro con correlación ítem total corregida inferiores a 0,30, previo a un análisis y contraste de la importancia del ítem en la revisión de la literatura. 

En la Tabla 2 se presentan los estadísticos descriptivos del instrumento ajustado de ocho ítems de prácticas preventivas de hantavirus; se observa que los ítems 2 y 3 presentaron valores de asimetría elevados superiores a 3 como valor absoluto y elevada curtosis. Los porcentajes de opciones de respuesta de los ítems mostraron en general puntuaciones altas, especialmente el ítem 8 que no registra puntuaciones bajas.

**Tabla 2:** Estadísticos descriptivos de los ítems.

Ítems	Porcentaje de opciones de respuesta					Propiedades métricas de los ítems			
	1	2	3	4	5	Media	DE	Asimetría	Curtosis
1	5,6	1,4	12,2	17,8	62,9	4,310	1.106	-1,714	2,243
2	1,9	0,9	3,3	9,4	84,5	4,737	0,737	-3,450	12,760
3	34,7	4,2	11,7	14,1	35,2	3,108	1,727	-0,163	-1,705
4	8,9	0,9	5,2	8,5	76,5	4,427	1,217	-2,102	3,045
5	1,4	0,5	4,7	7,5	85,9	4,761	0,690	-3,482	13,275
6	9,4	0,9	5,6	12,2	71,8	4,362	1,238	-1,945	2,491
7	12,7	1,4	8,0	12,7	65,3	4,164	1,383	-1,495	0,771
8	-	-	8,0	22,5	69,5	4,615	0,631	-1,405	0,805

DE: desviación estándar

En la Tabla 3 se presenta la matriz de correlaciones policóricas, la cual evidencia correlaciones bajas entre el ítem 11 y los ítems 3 y 4; el resto de los ítems presentan correlaciones cercanas a 0,30 o superiores a 0,40.

**Tabla 3:** Correlaciones policóricas inter-ítem.

	Correlación inter-ítem							
	Ítem 1	Ítem 2	Ítem 3	Ítem 4	Ítem 5	Ítem 6	Ítem 7	Ítem 8
Ítem 1	1,000							
Ítem 2	0,258	1,000						
Ítem 3	0,385	0,273	1,000					
Ítem 4	0,356	0,401	0,272	1,000				
Ítem 5	0,356	0,334	0,325	0,522	1,000			
Ítem 6	0,249	0,424	0,460	0,481	0,501	1,000		
Ítem 7	0,344	0,479	0,529	0,441	0,484	0,910	1,000	
Ítem 8	0,448	0,109	0,167	0,445	0,455	0,391	0,321	1,000

Se analizaron los índices de adecuación muestral, el valor de KMO fue de 0,7565 con un IC 95% de 0,709-0,820, la prueba de esfericidad de Bartlett fue de 833,5 (df = 28; p <0,001) y todos los ítems mostraron adecuación simple de la muestra superiores a 0,50, estas puntuaciones evidencian la viabilidad del AFE.

El método de mínimos cuadrados no ponderados y el análisis paralelo realizado en el programa FACTOR, sugieren que los ocho ítems subyacen a un solo factor, el cual explica el 60,70% de la varianza total de la prueba y las cargas factoriales oscilan entre 0,496 a 0,854 descritos en la Figura 1. Los resultados de las cargas factoriales son aceptables en base a los criterios de retener los ítems con cargas factoriales iguales o superiores a 0,40.

**Figura 1:** Modelo y cargas factoriales del instrumento de prácticas preventivas de hantavirus.

### Análisis de fiabilidad

El análisis de fiabilidad se calculó a través de la prueba de consistencia interna del alfa ordinal, utilizando las cargas factoriales de la matriz de correlación policóricas, esto dio como resultado 0,84, valor considerado como bueno. Cabe destacar que el valor de alfa ordinal es una medición de consistencia interna que se refiere a las puntuaciones obtenidas con el instrumento en la población y no una métrica del propio instrumento. 

## DISCUSIÓN

Este estudio instrumental se realizó con el objetivo de diseñar y validar un cuestionario de prácticas preventivas de hantavirus, en un contexto endémico, el cual incluyó la validez de contenido través de jueces expertos, prueba piloto, la validez de constructo a través de un análisis factorial exploratorio y la fiabilidad medida con el alfa ordinal.

En nuestro contexto no se identificaron muchos instrumentos que midan las practicas preventivas de hantavirus ^9^ ^-^ ^13^. De los estudios identificados, uno^ (^ ^9^ explica el uso de la revisión de la literatura y el material de prevención de hantavirus elaborado por las instituciones de salud para el diseño del cuestionario y otro^ (^ ^12^ reporta el uso de grupos focales para el diseño del cuestionario y la validación por un experto. Ninguno de los estudios presentó evidencias de validación psicométrica.

El cuestionario de prácticas preventivas de hantavirus aplicada en una comunidad endémica evidenció validez de contenido y valores de V de Aiken superiores 0,70 y un margen de error del 5%. El análisis factorial exploratorio muestra que los ocho ítems subyacen a un solo factor que explica el 60,70% de la varianza. La fiabilidad medida a través del alfa ordinal en base a las cargas factoriales de la matriz de correlación policóricas fue adecuada (0,84).

El instrumento diseñado y validado (material suplementario) es un aporte para medir las prácticas preventivas de hantavirus ya que podría aplicarse en la linea de base para el desarrollo de los programas de educación en salud para la prevención de hantavirus; además, podría contribuir a evaluar el cambio en las prácticas o comportamiento en estudios de pre y posintervenciones focalizadas. Este instrumento se diseñó en torno al principal enfoque de prevención disponible actualmente que incluye las prácticas de higiene en el hogar para evitar que el roedor ingrese a la vivienda y la limpieza segura de sus desechos ^15^ ^,^ ^16^, y la evidencia sobre la relación entre la presencia del roedor y el incremento de riesgo de los casos de hantavirus ^5^. Este diseño general del instrumento permite su aplicación en otros países del contexto latinoamericano, donde el hantavirus es endémico; sin embargo, es necesario tomar en cuenta la limitante de que en este cuestionario no fue posible medir los ítems relacionados con la siembra, cosecha y almacenamiento de granos en los predios cercanos a la vivienda, los cuales constituyen un riesgo para el contacto del ser humano con el vector y sus desechos.

Entre otras limitaciones del estudio podemos señalar el bajo porcentaje de respuesta obtenido en las preguntas sobre prácticas preventivas de hantavirus, relacionadas con la siembra, almacenamiento de la cosecha de productos agrícolas, que impidió que estos ítems fueran utilizados en el AFE, los cuales podrían permitir tener una lectura más completa de las prácticas preventivas de hantavirus; la selección de los participantes por muestreo por conveniencia no permite generalizar los resultados; no se verificó la estabilidad del instrumento en el tiempo, por lo que sería necesario en próximos estudios explorar la confiabilidad test-retest. Por último, dado que el cuestionario mide las prácticas preventivas por autorreporte, las respuestas podrían estar sesgadas por aprendizaje y autodefensa, ya que el participante tiende a contestar en base al conocimiento adquirido y a las respuestas esperadas. En consecuencia, en futuras investigaciones el cuestionario podría complementarse con evaluaciones cualitativas u otras técnicas de recolección de datos como la observación y el uso de listas de chequeo.

En conclusión, el cuestionario de prácticas preventivas de hantavirus es un instrumento unidimensional de ocho ítems, que mostró evidencias de validez de contenido, estructura y fiabilidad para medir las prácticas y/o actividades o conductas que realizan las personas en una comunidad endémica para prevenir el hantavirus.
